# Pragmatic Sensory Screening in Anorexia Nervosa and Associations with Autistic Traits

**DOI:** 10.3390/jcm9041182

**Published:** 2020-04-20

**Authors:** Emma Kinnaird, Yasemin Dandil, Zhuo Li, Katherine Smith, Caroline Pimblett, Rafiu Agbalaya, Catherine Stewart, Kate Tchanturia

**Affiliations:** 1 King’s College London, Department of Psychological Medicine, Institute of Psychology, Psychiatry and Neuroscience, London SE5 8AZ, UK; 2 National Eating Disorders Service, South London and Maudsley NHS Foundation Trust, London BR3 3BX, UK; 3 Child and Adolescent Eating Disorders Service, South London and Maudsley NHS Foundation Trust, London SE5 8AZ, UK; 4 Department of Psychology, Illia State University, Tbilisi 0162, Georgia

**Keywords:** anorexia nervosa, eating disorders, sensory sensitivity, autism

## Abstract

Background: Research suggests that people with anorexia nervosa (AN) experience subjective hypersensitivity to external sensations that may require consideration in treatment. These difficulties may be particularly pronounced in people with AN and high autistic traits. The purpose of this pilot study was to explore the use of a brief screening tool to assess sensory sensitivity in individuals receiving treatment for AN, and to assess if self-rated sensitivity in AN is related to autistic traits. Methods: 47 individuals receiving treatment for AN completed a brief sensory screening tool and self-rated their autistic traits. Individuals were also asked to give qualitative feedback on the screening tool. Results: People with AN and high autistic traits rated themselves as more hypersensitive compared to people with AN and low autistic traits. Feedback surrounding the use of the screener was positive. Conclusions: The results of this study suggest that the use of this screener may be beneficial in eating disorder settings to help adjust and calibrate treatment to personal needs, although further research and psychometric evaluation around the clinical use of the screener is required. The finding that people with AN and high autistic traits may experience elevated hypersensitivity also warrants further exploration in future research.

## 1. Introduction

Anorexia nervosa (AN) is an eating disorder (ED) characterised by the persistent restriction of energy intake, an intense fear of weight gain, and disturbance in the evaluation of weight and body shape [1]. With high levels of chronicity in this population and poor treatment response rates, there is an ongoing interest in how existing understandings of AN and related treatment approaches can be adapted [2,3,4]. One emerging research area is whether people with AN exhibit differences in how they experience their body and its relationship to the environment [5]. Research suggests that AN may be associated with dysregulated sensory processing of external stimuli. Questionnaire-based studies indicate that people with AN experience heightened sensory sensitivity (hypersensitivity) and are more likely to perceive these sensations as aversive, resulting in the attempted avoidance of sensory experiences [6,7,8,9]. This sensory profile has been associated with heightened levels of ED symptomatology, emotional dysregulation and body image disturbance, and appears to persist following treatment and weight restoration [7,8,9]. Sensory experiences, such as lower sensation seeking, are also linked to heightened feelings of self-disgust in this population [6]. It is therefore possible that the core symptom of food restriction in AN may in fact play a role in self-regulating distressing sensory experiences through avoidance. Alternatively, if people with AN have low tolerance of sensory signals, then this may limit their ability to use these sensations to inform their behaviour, or to self-regulate [8].

Whilst questionnaire-based studies consistently suggest hypersensitivity in AN, biological-based findings are more mixed. Recent systematic reviews of taste and smell experiments in this population have identified evidence for both hypersensitivity and hyposensitivity (lowered sensitivity) [10,11]. This apparent discrepancy between subjective and objective measures suggests that sensory dysregulation in AN may not only be driven by potential bottom-up alterations in biological sensitivity, but also by top-down processes in how this information is perceived, interpreted and integrated [5].

Sensory difficulties may be particularly central in understanding the presentation of AN in patients with high autistic traits. Autism is a neurodevelopmental disorder associated with difficulties in social communication, repetitive and/or restrictive behaviours and interests, and sensory problems [1]. Sensory difficulties are very common in autism, with around 95% of autistic adults self-reporting altered sensory processing [12]. Significantly around one in four people with AN present with high levels of autistic traits, and around one in ten people with AN meet criteria for an autism diagnosis [13,14,15]. Research suggests that autistic traits in AN may be associated with longer illness durations and poorer treatment outcomes, suggesting that treatment adaptations may be required. Recent qualitative research suggests that people with AN and high autistic traits may particularly benefit from adaptations addressing sensory difficulties associated with autism [16,17,18,19]. This research indicates that that sensory difficulties in autism may impact AN and its treatment in two key ways: firstly, food-related sensitivities such as an aversion to certain textures may motivate food avoidance [20,21,22]. Secondly, patients with high autistic traits may find ED service environments aversive: for example, an individual with hypersensitivity to sound may find loud treatment spaces overwhelming [20].

Therefore, people with AN- and people with AN and high autistic traits in particular- may benefit from an assessment of their sensory needs during treatment. For example, if an individual presents with a strong aversion to certain tastes, these could be addressed by working with a dietician to create a meal plan that adapts around these sensitivities. At present, sensory assessments in ED services are typically carried out by a trained occupational therapist. Existing self-report assessments of sensory sensitivity, such as the Adult Sensory Profile, are often lengthy and not always freely available [23]. In this context, clinicians treating this population could benefit from the use of a brief screening tool that assesses potential sensory difficulties. A screening approach could then be used to inform whether a more detailed assessment and treatment adaptations are required.

The aim of the current study was to pilot and explore the use and acceptability of a brief, pragmatic sensory screener in a national ED service. The secondary aim of the study was to explore whether self-rated sensory sensitivities in AN are related to autistic traits.

## 2. Experimental Section

### 2.1. Participants

Adult patients with a diagnosis of AN in the South London and Maudsley National Health Service (NHS) Foundation Trust National ED Service completed the measures as part of standard audit data collection in the service. Diagnoses of AN were made by trained clinicians upon admission to the treatment programme. A total of 47 patients completed all of the measures.

### 2.2. Materials

#### 2.2.1. Clinical and Demographic Information

Information on participant age, diagnosis, duration of ED, and body mass index (BMI) upon admission were taken from patient clinical notes.

#### 2.2.2. Brief Sensory Screener

A copy of the screener is located in the Supplementary Materials for this paper (Table S1). The development of the screener was based on the five basic senses: vision, hearing, smell, taste and touch. Other senses, such as interoception or proprioception, were not included to keep the measure accessible to individuals who may not be familiar with these other modalities. Participants were presented with visual scales for each sensory modality ranging from 0 (hyposensitive) to 10 (hypersensitive), and asked to indicate their sensory level on each scale. A score of 5 indicates no sensory differences. Examples of hyposensitivity and hypersensitivity are given for each modality, with hyposensitivity (under-sensitivity) and hypersensitivity (high sensitivity) defined at the beginning of the screener.

Following an initial pilot of the screener, clinicians and patients suggested that the “touch” modality was overly broad. They suggested that a screening tool for this population could benefit from separating wider domains of touch from food textures. Therefore, the final version includes separate rating scales for touch (with examples based on fabric textures) and texture (with examples based on food textures). A number of participants only completed the first version of the questionnaire, without the separate texture scale. These participants have been included in the current study, with variation in group numbers highlighted in the results.

#### 2.2.3. Item Autism Quotient (AQ-10)

The AQ-10 (adult version) is an autism screening tool recommended for use in adults with suspected high autistic traits [24]. It is a self-report tool consisting of 10 items, with a score of 6 and above indicating that an individual should be considered for a specialist autism assessment. This measure is widely used in this population, including for audit purposes [25,26].

#### 2.2.4. Hospital Anxiety and Depression Scale (HADS)

The HADS is a brief 14-item self-rating measure of anxiety and depression [27]. It consists of a subscale for anxiety, and a subscale for depression, with a score of 11 and above for each subscale indicating moderate to severe symptoms.

### 2.3. Procedure

Patients admitted to day-patient and inpatient ED services at the South London and Maudsley NHS Foundation Trust routinely complete the AQ-10 and HADS upon admission as part of standard audit data collection. Patients were additionally asked to complete the sensory screener, and to write down any feedback about the screener. This study was carried out as part of a clinical innovation project approved by the Clinical Governance and Audit Committee in South London and Maudsley NHS Trust (032019) in April 2019.

### 2.4. Analysis

Participants were divided into two groups depending on whether they scored above threshold on the AQ-10. Individuals scoring below threshold were classified as having low autistic traits (LAT), and individuals scoring above threshold were classified as having high autistic traits (HAT). Shapiro-Wilk tests confirmed that the sensory outcomes were normally distributed. Although sample sizes in each group were uneven, Levene’s tests suggested that the sample variances were equal on the sensory outcomes. Therefore, independent *t*-tests were used to compare groups on demographic and clinical characteristics. Categorical variables were compared using the chi square test. Sensory screener data were also analysed using independent *t*-tests. Cohen’s *d* was used to calculate effect sizes. Across the whole sample, relationships between sensory outcomes and autistic traits, anxiety and depression were explored using a regression analysis. The screener was evaluated using Cronbach’s alpha to calculate the internal consistency, and by soliciting written feedback from participants.

## 3. Results

### 3.1. Participant Characteristics

A total of 47 participants completed all measures. 30 participants scored below threshold on the AQ-10, forming the LAT group, and 17 participants scored above threshold to form the HAT group. Group clinical and demographic characteristics are summarised in Table 1. There were no significant differences between groups regarding their mean age, illness duration, HADS scores, or sex composition. By design the HAT group had significantly higher AQ-10 scores compared to the LAT group.

### 3.2. Sensory Screener

Group scores on the sensory screeners are summarised in Table 2. Patients with AN in the HAT group self-rated themselves as significantly more hypersensitive with medium-large effect sizes in the modalities of smell, vision, texture, and total screening scores, compared to LAT patients.

### 3.3. Associations with Clinical Variables

A regression analysis was performed using the full sample (*n* = 47) to explore the associations between sensory outcomes and related clinical variables (autistic traits, anxiety, and depression). Analyses suggested that higher autistic traits were associated with heightened sensitivity in the smell modality only (Table 3). Higher depression scores were associated with lower smell sensitivity. No other significant associations were identified.

### 3.4. Evaluation

Cronbach’s Alpha for the scale was 0.72, indicating acceptable internal reliability and that the individual items are measuring the same underlying concept. Across the sample, *n* = 9 (19.15%) of the participants gave feedback on the use of the screener. Feedback was generally positive, including that the form was “clear and easy to follow” and that it gave participants an opportunity to reflect on their sensory experiences. Participants felt that the screener was beneficial in highlighting a need for environmental adaptations:

“It can be very helpful to discover what a particular person likes or dislikes and will help to create an environment comfortable for people who suffer from eating disorders especially during meals.”

Negative feedback included changing the formatting and layout of the form to make it clearer, and concerns that only using rating scales did not leave the participants space to fully explore or explain their sensory sensitivities.

## 4. Discussion

The primary aim of the study was to explore the use and acceptability of a brief, pragmatic sensory screener in a national clinical ED service. This initial pilot study suggests that this screener could potentially be beneficial for use in ED treatment services, with participants generally giving positive feedback and the clinical team finding it helpful to work with this information in the context of treatment. However, further research in larger sample sizes, including an investigation of its psychometric properties, is needed to establish the utility of this screening tool. Potential benefits to this screener identified in this pilot study that could be explored in future research are that it may help both patients and their clinician with awareness, recognition and reflection surrounding sensory difficulties, and their implications for formulation and treatment. The nursing team and dieticians also reported that the tool was quick and easy to administer, and gives useful information that could help make treatment more tailored to individual needs and personalise treatment strategies.

The screener does not provide a detailed exploration of the individual’s sensory sensitivities: for example, the single scales for each sensory modality do not capture if someone experiences both hyper- and hyposensitivity in certain situations. Rather, the screener appears to help stimulate thought and discussion around individual sensory needs, and highlights where assessment by an occupational therapist, or using a more detailed sensory measure, could be beneficial. To our knowledge this is the first development of a sensory screening tool specifically for use in ED populations. The piloting of this tool suggests that measures for use in this population could benefit from distinguishing between general sensory sensitivities, and food-specific sensitivities. For example, clinicians and patients recommended that food texture sensitivity be measured independently from general touch/texture sensitivity, and the results indicate that participants did indeed rate themselves differently on these separated modalities.

The secondary aim of the study was to explore whether self-rated sensory sensitivities in AN are related to autistic features, finding that people with AN and high autistic traits scored themselves as more sensitive in the areas of smell, vision, texture, and overall total screening scores, compared to participants with low autistic traits. This is the first study to explore the relationship between self-rated sensory sensitivity and autistic traits in people with AN, and suggests that autistic traits may contribute to hypersensitivity in this condition [6,7,8,9]. The results of this study strongly indicate that future research in this area should consider the potential role of autistic traits in study design and analysis. The finding also supports previous qualitative research in this area suggesting that people with AN and high autistic traits may indeed experience elevated sensory difficulties, and reinforces the possibility that this population may benefit from an assessment of their sensory needs during treatment, and subsequent environmental and dietary adaptations as appropriate [20,22].

However, the nature of the relationship between autistic traits, AN, and sensory sensitivities remains unclear. In the current study, people with AN and high autistic traits had higher scores compared to those with low autistic traits. However, the regression analysis suggested that autistic traits predicted elevated sensitivity in the area of smell only. It is possible that autistic traits impact sensory sensitivity in AN through an additional mediating variable, although a strength of this study is that it controlled for the potential confounders of anxiety and depression in the regression analysis. Two prior studies have explored objective experimental measures of smell sensitivity and autistic traits in AN with conflicting results [28,29]. This reflects evidence from previous neuropsychological research in AN which has found a similar lack of agreement between self-report and experimental measures of cognitive flexibility [30]. It is likely that future research in AN could benefit from using both types of approaches. A key advantage of using self-report measures in clinical settings is that clinicians can more easily carry out subjective reports and use this information to tailor treatment approaches, compared to experimental measures which may need additional resources and expertise. Further research in this area is needed to explore associations between self-reported sensory sensitivity and autistic traits in AN, particularly relation to potential underlying mechanisms. Future research could explore mechanisms hypothesised to influence sensory processing in autism in AN to illuminate the relationship between these areas. In particular, sensory processing in autism has been hypothesised to be related to biased central coherence in this population: autistic people are theorised to exhibit a bias towards detail-orientated information processing as opposed to global processing, or seeing the “bigger picture”, which may contribute to hypersensitivity [31]. People with AN also exhibit a bias towards detail-orientated processing which has previously been linked to altered visual processing in this condition [32,33,34,35]. Further research on sensory processing and autistic traits in AN could consider this as a potential underlying mechanism.

There are a number of limitations to this study. The nature of this pilot and feasibility study meant that the sample size is relatively small, and future studies could benefit from including a higher number of participants. In particular, the current study did not include a healthy control comparison group. Therefore, it cannot draw conclusions surrounding whether people with AN rate their sensory sensitivity levels differently compared to healthy controls. Findings of hypersensitivity in this study therefore reflect people with AN rating themselves as highly sensitive on the screening measure (against a control marker of “no sensory differences”), rather than people with AN rating themselves as highly sensitive in comparison to people without the condition.

In addition, the current study did not include a full evaluation of the psychometric qualities of the screener, although a preliminary assessment does indicate acceptable internal reliability. The sensory screener was designed as a brief, pragmatic measure for use in clinical practice. It was not designed as a research tool, and therefore the goal of the current study was to explore its use and utility, rather than establishing its reliability and validity, or its agreement with other measures of sensory sensitivity. As the findings of the current study suggest that such a screening tool may be clinically beneficial both to patient and clinicians in ED services, future research could further explore the development and psychometric validation of sensory measures and screening in this population. The development of this sensory screener, in particular separating the modality of touch into a non-food example and a food-based “texture” example, suggests that research on the use and development of sensory screening in this population should consider whether sensory difficulties in AN are specific to food-related sensations or more generalised. For clinicians, in addition to the use of screening tools a more detailed assessment of sensory difficulties could consider using other tools, such as the Adult Sensory Profile, or the Swedish Eating Assessment for Autism Spectrum Disorders [23,36]. This assessment explores the presence of eating difficulties associated with autism, including items related to sensory sensitivities.

Finally, the present study does present an initial exploration of the relationship between subjective sensory sensitivity and autistic traits within AN populations, but does so in relation to self-rated autistic traits only. The AQ-10 was used in this study to distinguish people with high autistic traits as it is currently recommended for use in healthcare services for this purpose by UK clinical guidelines [37]. However, the AQ-10 and the original 50-item AQ may lack efficacy in distinguishing autism cases in clinical populations [38,39,40,41]. In the current study, that the AQ-10 may lack accuracy is suggested by the fact that participants in the HAT group had a lower, albeit non-significant, duration of illness compared the LAT group, whereas characteristics associated with autism assessed with experimental measures are associated with longer illness durations in AN [17]. Future research in this area should consider exploring sensory sensitivity in individuals with AN only compared to people with AN and a diagnosis of autism, or using gold-standard autism measures such as the Autism Diagnostic Observation Schedule (ADOS) known to be effective in this population [42]. In addition, future explorations of sensory sensitivities in AN and autism could benefit from also including a sample group of autistic people without AN.

In conclusion, the findings of the current study indicate the potential utility of using a brief sensory screener to evaluate subjective sensory sensitivity in individuals accessing ED treatment. In addition, the study suggests that subjective hypersensitivity in AN may be related to autistic traits. Implications for future research and potential clinical adaptations are discussed.

## Figures and Tables

**Table 1:** Summary of group differences on clinical and demographic characteristics.

	LAT (*n* = 30)	HAT (*n* = 17)	*t*-Test (*df* = 45)	*p*	*d*
Age (Years)	30.23 (9.60)	27.76 (10.08)	0.83	0.410	0.25
Sex	96.67% female	88.24% female	*X*^2^ = 1.29	0.256	-
AN Subtype	93.33% AN-R 6.67% AN-BP	93.75% AN-R 6.25% AN-BP	*X*^2^ = 0.03	0.957	-
Illness duration (Years)	11.08 (8.77)	8.73 (9.32)	0.76	0.451	0.26
BMI	14.41 (2.02)	14.55 (1.67)	−0.23	0.821	0.07
AQ-10	3.60 (1.40)	7.59 (1.12)	−10.02	<0.001	3.04
HADS Anxiety	13.97 (4.70)	13.71 (6.69)	0.015	0.878	0.05
HADS Depression	11.21 (4.69)	11.82 (5.04)	−0.42	0.677	0.13

Group differences are presented as group means, with standard deviations in parentheses. Low autistic traits group (LAT), high autistic traits group (HAT), degrees of freedom (df), anorexia nervosa (AN), restrictive subtype (AN-R), binge-purge subtype (AN-BP), Adult Autism Quotient 10 item (AQ-10), Hospital Anxiety and Depression Scale (HADS), cohen’s *d* (*d*), chi square (*X*^2^).

**Table 2:** Summary of group differences on sensory screening scores.

	LAT (*n* = 30)	HAT (*n* = 17)	*t*-Test (*df* = 45)	*p*	*d*
Taste	5.23 (2.25)	5.91 (2.39)	−0.97	0.337	0.29
Smell	5.67 (2.31)	7.65 (2.57)	−2.71	0.010	0.82
Vision	5.67 (2.30)	7.18 (2.30)	−2.36	0.022	0.72
Sound	6.13 (2.56)	7.18 (3.15)	−1.24	0.223	0.38
Touch	5.60 (2.34)	6.24 (2.86)	−0.82	0.414	0.25
Texture	5.44 (2.12) *n* = 18	7.31 (2.63) *n* = 16	−2.29	0.029	0.79
Total Without Texture	28.23 (6.98)	35.06 (9.52)	−2.82	0.007	0.86
Total With Texture	32.94 (8.63) *n* = 18	41.5 (10.31) *n* = 16	−2.63	0.013	0.90

Group differences are presented as group means, with standard deviations in parentheses. Abbreviations: low autistic traits group (LAT), high autistic traits group (HAT), degrees of freedom (df).

**Table 3:** Regression analysis.

	AQ-10 (*B*, *t*, *p*)	HADS Anxiety (*B*, *t*, *p*)	HADS Depression (*B*, *t*, *p*)
**Taste**	0.17, 1.05, 0.299	−0.03, −0.35, 0.726	−0.08, −0.92, 0.361
**Smell**	0.43, 2.57, 0.014	0.10, 1.36, 0.181	−0.25, −2.70, 0.010
**Vision**	0.22, 1.43, 0.161	−0.01, −0.21, 0.838	0.08, 0.96, 0.343
**Sound**	0.12, 0.59, 0.559	−0.01, −0.08, 0.938	0.02, 0.16, 0.871
**Touch**	0.04, 0.24, 0.814	0.03, 0.31, 0.760	−0.00, −0.04, 0.966
**Texture**	0.34, 1.59, 0.123	−0.02, −0.19, 0.854	−0.13, −1.17, 0.251
**Total Without texture**	1.10, 1.87, 0.069	0.13, 0.49, 0.624	−0.22, −0.69, 0.496
**Total With texture**	1.48, 1.67, 0.106	0.13, 0.38, 0.704	−0.45, −0.95, 0.349

Abbreviations: low autistic traits group (LAT), high autistic traits group (HAT), Adult Autism Quotient 10 item (AQ-10), Hospital Anxiety and Depression Scale (HADS), regression coefficient (*B*), regression t statistic (*t*), *p*-value (*p*).

## Supplementary Materials

The following are available online at https://www.mdpi.com/2077-0383/9/4/1182/s1, Table S1: Brief Sensory Screener.
