# Supplementary material for: Pragmatic Sensory Screening in Anorexia Nervosa and Associations with Autistic Traits
Source: J Clin Med. 2020 Apr 20;9(4):1182. doi: 10.3390/jcm9041182 (PMC7230430; doi:10.3390/jcm9041182)
Supplement: Supplementary file 1 [file jcm-09-01182-s001.pdf]

PEACE

Pathway for Eating disorders and Autism developed from Clinical Experience

Sensory Summary

Mark where you think you are on the below scales. Hypersensitivity means you are highly sensitive to sensations and may try and avoid them where possible; hyposensitivity means you have lower sensitivity and may try to seek out these sensations. There are examples below each scale. If you think you are neither hyper/hyposensitive and have no sensory differences, mark yourself in the middle as a 5.

Taste

0	1	2	3	4	5	6	7	8	9	10

(Hyposensitive) (No sensory differences) (Hypersensitive)

If I am hyposensitive, I might add lots of salt to my food to make it taste stronger. If I am hypersensitive, I might prefer to eat bland foods as I find them too strong.

Smell

0	1	2	3	4	5	6	7	8	9	10

(Hyposensitive) (No sensory differences) (Hypersensitive)

If I am hyposensitive, I might not notice strong smells and enjoy smelling essential oils. If I am hypersensitive, I might dislike smelly places like a canteen and find smells overpowering.

Vision

0	1	2	3	4	5	6	7	8	9	10

(Hyposensitive) (No sensory differences) (Hypersensitive)

If I am hyposensitive, I might really like watching bright light displays. If I am hypersensitive, I might prefer to have lights dimmed or turned off.

Sound

0	1	2	3	4	5	6	7	8	9	10

(Hyposensitive) (No sensory differences) (Hypersensitive)

If I am hyposensitive, I might turn my music up loud and dislike silence. If I am hypersensitive, I might dislike loud spaces and put my hands over my ears.

Touch

0	1	2	3	4	5	6	7	8	9	10

(Hyposensitive) (No sensory differences) (Hypersensitive)

If I am hyposensitive, I might enjoy rubbing my hands on soft fabric or a soft toy. If I am hypersensitive, I might dislike and avoid touching certain fabrics.

Texture

0	1	2	3	4	5	6	7	8	9	10

(Hyposensitive) (No sensory differences) (Hypersensitive)

If I am hyposensitive, I might really enjoy the feeling of certain food textures in my mouth (such as liking crunchy food). If I am hypersensitive, I might strongly dislike and avoid eating certain food textures (such as mashed potato).
